# Distance makes a difference in crystalline photoluminescence

**DOI:** 10.1038/s41467-020-19377-6

**Published:** 2020-11-04

**Authors:** Zibao Gan, Yungui Liu, Lin Wang, Shuqing Jiang, Nan Xia, Zhipeng Yan, Xiang Wu, Junran Zhang, Wanmiao Gu, Lizhong He, Jingwu Dong, Xuedan Ma, Jaeyong Kim, Zhongyan Wu, Yixuan Xu, Yanchun Li, Zhikun Wu

**Affiliations:** 1 Key Laboratory of Materials Physics, Anhui Key Laboratory of Nanomaterials and Nanotechnology, CAS Center for Excellence in Nanoscience, Institute of Solid State Physics, Chinese Academy of Sciences, 230031 Hefei, China; 2 Institute of Physical Science and Information Technology, Anhui University, 230601 Hefei, China; 3 State Key Laboratory of Geological Processes and Mineral Resources, China University of Geosciences, 430074 Wuhan, China; 4 Center for High Pressure Science and Technology Advanced Research, 201203 Shanghai, China; 5 Center for High Pressure Science (CHiPS), State Key Laboratory of Metastable Materials Science and Technology, Yanshan University, Qinhuangdao, 066004 Hebei, China; 6 Multidiscipline Research Center, Institute of High Energy Physics, Chinese Academy of Sciences, 100049 Beijing, China; 7 China University of Chinese Academy of Sciences, 100049 Beijing, China; 8 Centre for Nanoscale Materials, Argonne National Laboratory, 9700 South CassAvenue, Lemont, IL 60439 USA; 9 HYU-HPSTAR-CIS High Pressure Research Center, Department of Physics, Hanyang University, Seoul, 04763 Republic of Korea

**Keywords:** Optical materials, Nanoparticles, Chemical physics

## Abstract

Crystallization-induced photoluminescence weakening was recently revealed in ultrasmall metal nanoparticles. However, the fundamentals of the phenomenon are not understood yet. By obtaining conformational isomer crystals of gold nanoclusters, we investigate crystallization-induced photoluminescence weakening and reveal that the shortening of interparticle distance decreases photoluminescence, which is further supported by high-pressure photoluminescence experiments. To interpret this, we propose a distance-dependent non-radiative transfer model of excitation electrons and support it with additional theoretical and experimental results. This model can also explain both aggregation-induced quenching and aggregation-induced emission phenomena. This work improves our understanding of aggregated-state photoluminescence, contributes to the concept of conformational isomerism in nanoclusters, and demonstrates the utility of high pressure studies in nanochemistry.

## Abstract (web summary)

Crystallization-induced photoluminescence weakening in ultrasmall metal nanoparticles is not well understood. Here, the authors study conformational isomers of gold nanoclusters to examine the effect of interparticle distance on this phenomenon, and propose a model of distance-dependent non-radiative excitation electron transfer.

## Introduction

Photoluminescence (PL) is a very intriguing phenomenon, and it has gained extensive attention for many years^1–5^. However, the understanding of aggregated state PL mechanism is far from completeness. Two well-known phenomena, aggregation-caused quenching (ACQ)^6–8^ and aggregation-induced emission (AIE)^8–10^, were observed in solid materials. Comparable to the crystallization-induced quenching^11–14^ or concentration quenching of conventional luminogens^11,15–17^, a crystallization-induced PL weakening (CIPW) phenomenon (that is, the crystalline PL is less extensive than the amorphous PL) was recently observed in metal nanoclusters (NCs)^18,19^. To understand the fundamentals, investigating the conformational isomer crystal PL might be helpful since this kind of investigation can provide some insight into the structure-PL correlation. However, unfortunately, conformational isomer crystals for such an investigation are not accessible to us. Conformational isomerism is not trivial, and it is known that the conformation change in biological macromolecules can result in dramatic function differences (e.g., toxic vs*.* nontoxic)^20^. For inorganic (or inorganic-organic hybrid) nanoparticles (NPs), the concept of conformational isomerism has not been introduced until now, although it was indicated by some experimental or theoretical results^21,22^. The primary challenge for conformational isomerism research in inorganic (or inorganic-organic hybrid) NPs lies in the precise determination of NPs’ conformations, especially for relatively large NPs. Recent progress in ultrasmall noble metal NPs (often called NCs) has opened up exciting opportunities for isomerism research at the nanoscale since their compositions and structures (including conformations) can be precisely tuned and determined^23–33^. Indeed, the structural isomers in gold NCs^34^ has been experimentally revealed by Tian, et al.^35^, which also inspired our enthusiasm to search for conformational isomerism in metal NCs. Very recently, Pradeep et al. reported the quasi-conformational isomerism of [Ag_29_(BDT)_12_(TPP)_4_]^3−^ in two different polymorphic forms by X-ray crystallography, cubic and trigonal, which were obtained by regulating the solvent for crystallization^36^. However, it is not definite yet whether they are strictly conformational isomers since the complete formula (including the counter ions) was not given in either case^36,37^. An important implication of the mentioned work is that the introduction of the second ligand may give rise to the conformational isomerization of metal NCs because the second ligand provides diverse inter(intra)-NP interactions and influences the symmetry of the ligand shell. Nevertheless, it is worth noting that the introduction of the second ligand may also increase the difficulty of crystallization due to the possible increase in the flexibility or entropy of metal NCs^38^. The bare sulfur as the second ligand could be a good choice after balancing different influencing factors because bare sulfur has limited flexibility.

We recently successfully crystallized the sulfur-SCH_2_Ph mixture-protected gold NCs (e.g., Au_60_S_6_(SCH_2_Ph)_36_ and Au_60_S_7_(SCH_2_Ph)_36_) at high quality^18,39^. Motivated by this, here we introduce a single sulfur to an Au_60_S_7_(SCH_2_Ph)_36_ (abbreviated as Au_60_S_7_) NC. We obtain conformational isomers of gold NCs and investigate the crystalline PL in depth.

## Results

### Synthesis and characterization

The synthesis is simple and refers to our previous surface single-atom tailoring method^39^. Briefly, the Au_60_S_7_ NCs were etched with excess HSCH_2_Ph at 100 °C overnight, and the target NCs were isolated by preparative thin-layer chromatography (PTLC) (see “Methods” section for details). Single crystals of the purified NCs were fostered by the vapor diffusion of acetonitrile into the benzene solution of the purified NCs at 5 °C, and two types of crystals (rectangular and needle-like crystals) were concurrently obtained after one month (Supplementary Fig. 1). It should be noted that acetonitrile is a critical solvent to yield needle-like or rectangular crystals. Without acetonitrile, no any crystal was obtained (Supplementary Fig. 2a) even if the culture solutions were fostered for a longer time (e.g., three months). With the introduction of acetonitrile into the benzene solution (1/1, V/V), low-quality needle-like crystals were observed but no rectangular crystals appeared (Supplementary Fig. 2b). However, more needle-like crystals in high quality were found and a few rectangular crystals emerged (Supplementary Fig. 1a) when the ratio of acetonitrile to benzene was increased to 2/1 (V/V). With the ratio further increased (4/1, V/V), a large number of rectangular crystals with high quality were yielded (Supplementary Fig. 1b). Thus, the type, content and even quality of the crystals are closely related to the acetonitrile content in the mixture solvent. One possible explanation is that the Au-philic, polar CH_3_CN promotes and influences the self-assembly of Au_60_S_8_ clusters in the crystals. High content of CH_3_CN facilitates the forming of relatively high polar rectangular crystals, while low content of CH_3_CN benefits the yielding of relatively low polar needle-like crystals.

The molecular compositions of the two types of crystals were characterized by electrospray ionization mass spectrometry (ESI-MS). No signal was observed in either positive or negative mode without the addition of cesium acetate (CsOAc), indicating their charge neutrality. To impart charges, CsOAc was added to their solution to form positively charged [cluster+xCs]^x+^ adducts in the electrospray process. As shown in Supplementary Fig. 3, two intense peaks at mass/charge ratios (*m/z*) 8387.03 and 5635.57 were observed in the ESI-MS of the rectangular and needle-like crystals, respectively, which can be readily assigned to [Au_60_S_8_(SCH_2_Ph)_36_Cs_2_]^2+^ (calculated: 8387.68, deviation: 0.65) and [Au_60_S_8_(SCH_2_Ph)_36_Cs_3_]^3+^ (calculated: 5636.09, deviation: 0.52), respectively. Thus, the NCs in the rectangular and needle-like crystals should have the same composition, Au_60_S_8_(SCH_2_Ph)_36_, which was further verified by the following single-crystal X-ray crystallography (SCXC) analysis.

### Crystal structures with double isomerism

**Fig. 1:**
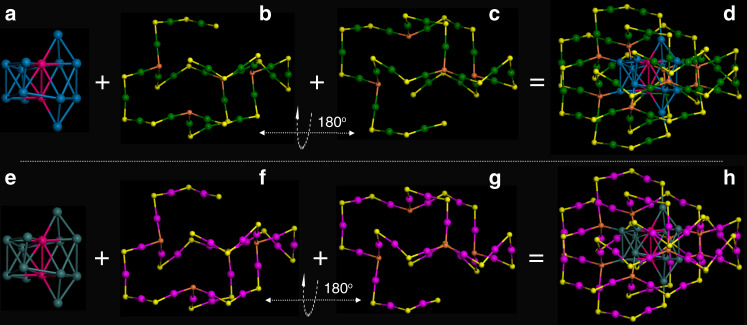
The anatomy of Au_60_S_8*r*_ and Au_60_S_8*n*_. (**a**) Au_14_ kernel, (**b**) Au_23_S_4_(SCH_2_Ph)_18_ motif, (**c**) Au_23_S_4_(SCH_2_Ph)_18_ motif, (**d**) the overall Au_60_S_8*r*_ framework; (**e**) Au_14_ kernel, (**f**) Au_23_S_4_(SCH_2_Ph)_18_ motif, (**g**) Au_23_S_4_(SCH_2_Ph)_18_ motif, and (**h**) the overall Au_60_S_8*n*_ framework. Note: carbon and hydrogen atoms are omitted; Color labels: the *μ*_4_-S atoms in orange, other S atoms in yellow, the kernel and staple gold atoms in different colors (To differentiate between Au_60_S_8*r*_ and Au_60_S_8*n*_, the kernel and staple gold atoms are shown in different colors, except that two gold atoms in the kernel are shown in red).

**Fig. 2:**
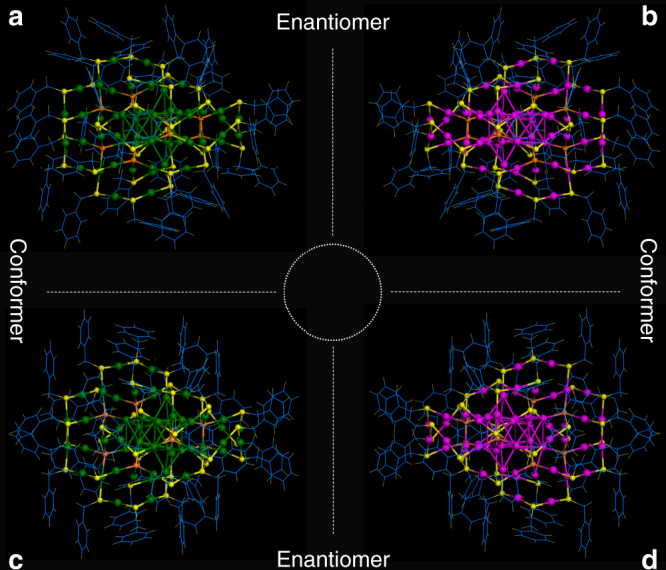
The overall structures of Au_60_S_8*r*_ and Au_60_S_8*n*_ NCs. (**a**) One Au_60_S_8*r*_ NC extracted from the Au_60_S_8*r*_ crystal, (**b**) another extracted Au_60_S_8*r*_ NC being the enantiomer of **a**, (**c**) one Au_60_S_8*n*_ NC extracted from the Au_60_S_8*n*_ crystal, and (**d**) another extracted Au_60_S_8*n*_ NC being the enantiomer of **c**. Color labels: the *μ*_4_-S atoms in orange, other S atoms in yellow, the gold atoms in enantiomers are respectively shown in green and magenta.

**Fig. 3:**
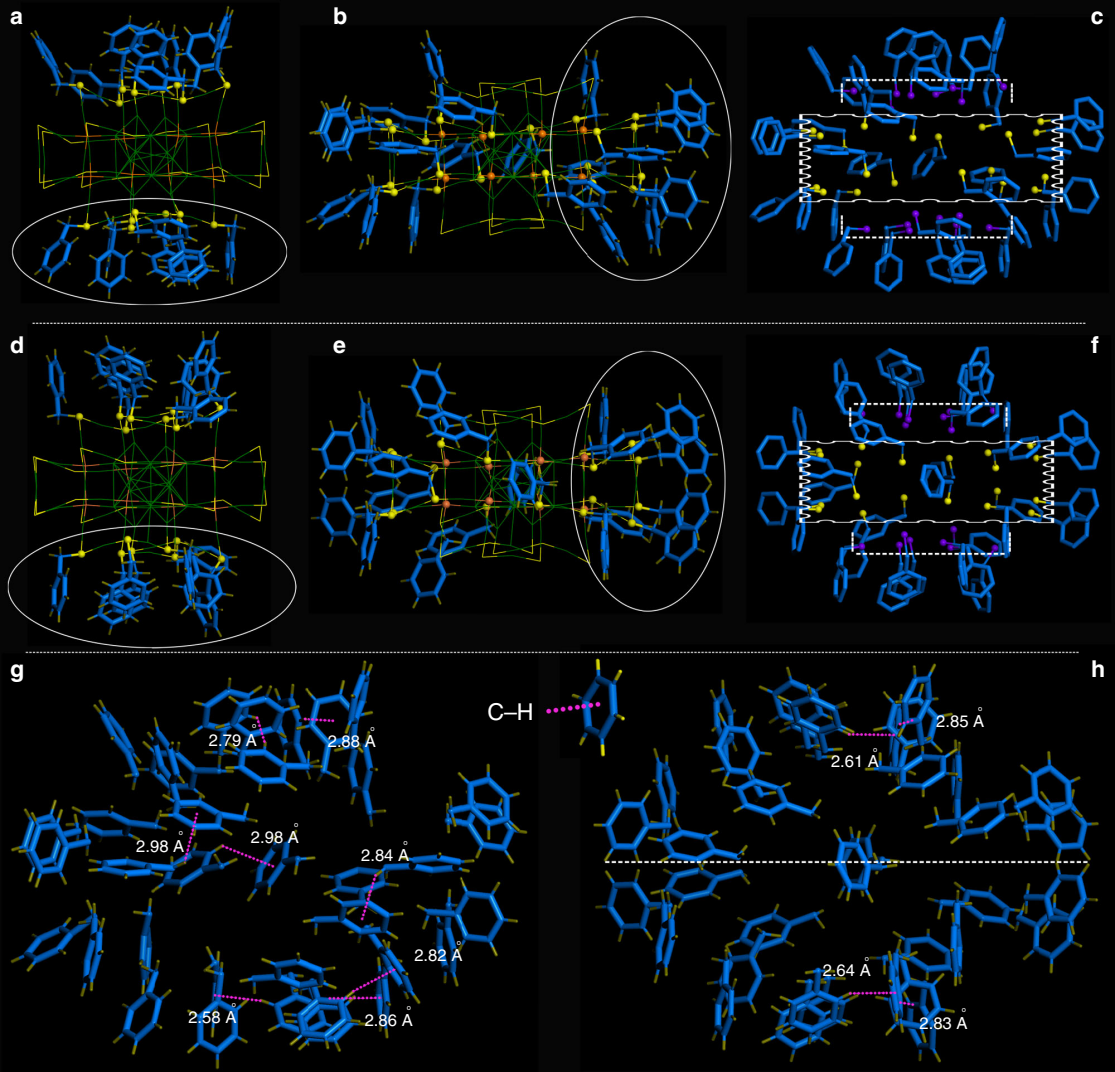
The assembly of phenylmethanethiolates on Au_60_S_8*r*_ and Au_60_S_8*n*_ surfaces. (**a**) Highlight of the distribution of phenylmethanethiolates on both sides of Au_60_S_8*r*_, (**b**) highlight of the distribution of phenylmethanethiolates in the middle section of Au_60_S_8*r*_, (**c**) illustration of the surface assembly of phenylmethanethiolates in another view for Au_60_S_8*r*_, (**d**) highlight of the distribution of phenylmethanethiolates on both sides of Au_60_S_8*n*_, (**e**) highlight of the distribution of phenylmethanethiolates in the middle section of Au_60_S_8*n*_, (**f**) illustration of the surface patterns of phenylmethanethiolates in another view for Au_60_S_8*n*_, (**g**) illustration of the intraparticle C–H···π interactions on the surface of Au_60_S_8*r*_, and (**h**) illustration of the intraparticle C–H···π interactions on the surface of Au_60_S_8*n*_ (for clarity, sulfur atoms are shown in purple and yellow and hydrogen atoms are omitted in **c** and **f**).

The Au_60_S_8_(SCH_2_Ph)_36_ (Au_60_S_8*r*_ for short) NCs in the rectangular crystal crystallized in a P1/2c space group, while the Au_60_S_8_(SCH_2_Ph)_36_ (Au_60_S_8*n*_ for short) NCs in the needle-like crystal crystallized in a C2/c space group. Both types of crystals have two pairs of chiral Au_60_S_8_(SCH_2_Ph)_36_ NCs in the unit cell, as shown in Supplementary Fig. 4. The anatomy of structures demonstrates that both Au_60_S_8*r*_ and Au_60_S_8*n*_ are composed of an Au_14_ kernel protected by a pair of Au_23_S_4_(SCH_2_Ph)_18_ staples (Fig. 1). The Au_14_ kernel can be viewed as two bi-tetrahedral Au_8_ units with an *fcc*-based antiprismatic shape sharing two gold atoms (Fig. 1a, e), and the Au_23_S_4_(SCH_2_Ph)_18_ staple can convert to the other one by rotating 180° along the C_2_ symmetry axis (Fig. 1b, c, f, g). Therefore, Au_60_S_8*r*_ and Au_60_S_8*n*_ NCs have no obvious differences in the framework structure, which is also supported by their negligible bond length and angle differences in the shells of Au_60_S_8*r*_ and Au_60_S_8*n*_ NCs (e.g., average Au-S bond: 2.306 vs. 2.310 Å; average S–Au–S bond angle: 172.8° vs. 172.6°, respectively). To clarify this, a pair of enantiomers are extracted from the Au_60_S_8*r*_ (Fig. 2a, b) and Au_60_S_8*n*_ (Fig. 2c, d) crystals. As shown in Fig. 2a, c, b, d, although there are no detectable differences in their framework structures, the assembly patterns of phenylmethanethiolate on the NC surfaces are obviously different. In other words, the surface ligands on the two NCs have different conformations. Therefore, Au_60_S_8*r*_ and Au_60_S_8*n*_ NCs are conformational isomers (conformer). As mentioned above, Au_60_S_8*r*_ and Au_60_S_8*n*_ NCs are racemic mixtures, and the chirality originates not only from the arrangement of phenylmethanethiolate (Fig. 2a, b, c, d) but also from the packing of both the Au_23_S_4_(SCH_2_Ph)_18_ staples and Au_14_ kernel (Supplementary Fig. 5, Au_60_S_8*r*_ as an example). For an illustration of double isomerism, see Fig. 2a–c. Such a double isomerism phenomenon was not previously reported in nanochemistry to the best of our knowledge. The phenylmethanethiolates nonuniformly distribute on the NC surface (Fig. 3a, b, d, e): 18 of them distribute in the middle section of the NC, which appears to be the torso of a millipede, and the left 18 phenylmethanethiolates dispersedly distribute on both sides similar to the feet of the millipede (Fig. 3c, f). Obviously, the phenylmethanethiolate ligands (highlighted by white ellipses in Fig. 3a, d, b, e) on the Au_60_S_8*r*_ and Au_60_S_8*n*_ surfaces have different stereochemical structures or occupied sites (also see Supplementary Figs. 6, 7). There are various possible intraparticle C–H···π interactions since the distance between the C-H and the closest benzene ring ranges from 2.58 to 2.98 Å (averaged: 2.78 ± 0.20 Å) in Au_60_S_8*r*_ and from 2.61 to 2.85 Å (averaged: 2.73 ± 0.12 Å) in Au_60_S_8*n*_^40,41^. Moreover, the so-called C–H···π interactions in Au_60_S_8*r*_ are randomly distributed, which is obviously different from those in Au_60_S_8*n*_ with approximate plane symmetrical structures of the phenylmethanethiolate assembly (see Fig. 3g, h).

**Fig. 4:**
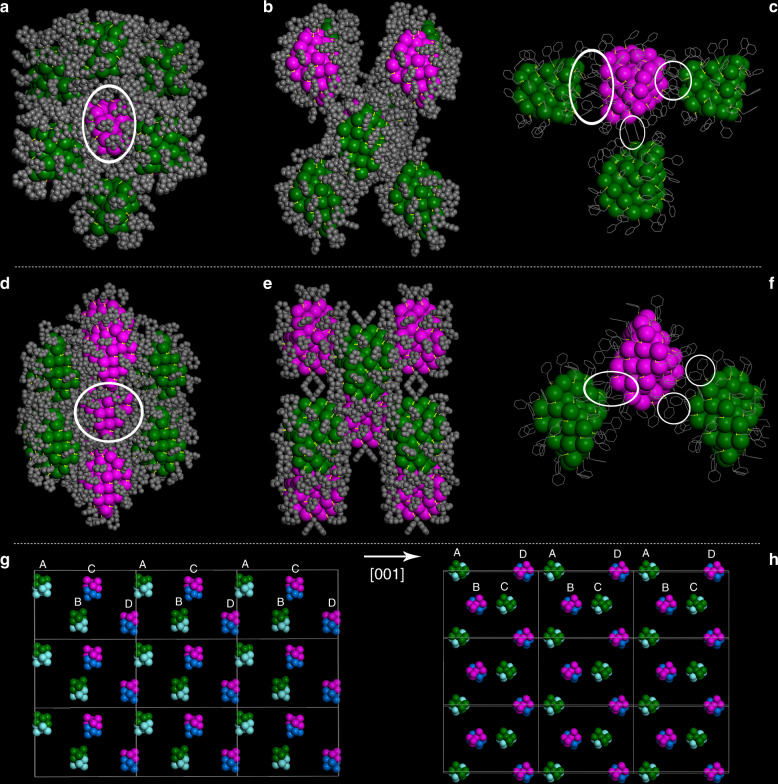
The interparticle self-assembly and crystallographic arrangement of Au_60_S_8*r*_ and Au_60_S_8*n*_. (**a**, **b**) Coordination environment of Au_60_S_8*r*_ in the crystal: front view (**a**) and side view (**b**); (**c**) the symmetry-matching of the contacting ligands in the Au_60_S_8*r*_ crystal; (**d**, **e**) coordination environment of Au_60_S_8*n*_ in the crystal: front view (**d**) and side view (**e**); (**f**) the symmetry-matching of the contacting ligands in the Au_60_S_8*n*_ crystal; (**g**, **h**) the crystallographic arrangement of Au_60_S_8*r*_ (**g**) and Au_60_S_8*n*_ (**h**) in the unit cells (note: hydrogen atoms are omitted in **a**–**f**, to facilitate the observation of crystallographic arrangement, the NCs are replaced by their chiral kernels in **g**, **h**).

Au_60_S_8*r*_ and Au_60_S_8*n*_ also have different interparticle interactions and crystallographic arrangements. As shown in Fig. 4, the central NC (highlighted by a white circle in Fig. 4a) in the Au_60_S_8*r*_ crystal has eight near neighbors, two of which have the same chirality as the central one (Fig. 4b), while the left six have the enantiomorphous chirality. In the Au_60_S_8*n*_ crystal, the central NC (highlighted by a white circle in Fig. 4d) has ten close neighbors, four of which have the same chirality as the central one (Fig. 4e), while the left six have the opposite chirality. The stable and tight assembly among the NCs should be related to the reported symmetry-matching of the contacting ligands^40^. Careful inspection also reveals that the phenylmethanethiolates resemble the tooth of a gear, by which the neighboring NCs firmly interlock in both Au_60_S_8*r*_ and Au_60_S_8*n*_ crystals (Fig. 4c, f). However, the interparticle C–H···π and π···π interactions were detected in Au_60_S_8*r*_ and Au_60_S_8*n*_ crystals, respectively (see Supplementary Fig. 4), which might result in different crystallographic arrangements, although they adopt similar stacking sequences of “ABCD” in the crystals (Fig. 4g, h). In Au_60_S_8*r*_, the stacking layers with the same chiralities are continuously arranged along the [001] direction (i.e., NCs in the “A” stacking layer have the same chirality with NCs in the “B” layer but are different from NCs in the “C” and “D” layers); however, in Au_60_S_8*n*_, the stacking layers with different chiralities are alternately arranged along the [001] direction (i.e., NCs in the “A” and “C” stacking layers have the same chirality but are different from NCs in the “B” and “D” layers). The abovementioned analyses unambiguously demonstrate that Au_60_S_8*r*_ and Au_60_S_8*n*_ are conformational isomers. It is worth noting that the concept of conformational isomerism has not been previously introduced in the field of nanochemistry to the best of our knowledge. Interestingly, the two conformational isomers can be converted to each other by controlling the crystallization solvent. Specifically, when Au_60_S_8*r*_ was dissolved and fostered in the mixture of 3 ml of benzene and 6 ml of acetonitrile, after one month, needle-like crystals were obtained (Supplementary Fig. 8), which were identified to be Au_60_S_8*n*_ by UV/vis/NIR, PL and PTLC (Supplementary Figs. 9–11). However, when Au_60_S_8*n*_ was dissolved and fostered in the system of 3 ml of benzene and 12 mL of acetonitrile, one month later, rectangular crystals were observed (Supplementary Fig. 12). The rectangular crystals were verified to be Au_60_S_8*r*_ by multiple characterizations, as shown in Supplementary Figs. 13–15. Thus, the conversion between the two conformational isomers again demonstrates the importance of acetonitrile in crystallizing the Au_60_S_8_ NCs: high-content of acetonitrile is helpful to the growth of relatively high polar Au_60_S_8*r*_ crystals, while low content of CH_3_CN is beneficial to the forming of low polar Au_60_S_8*n*_ crystals.

### Crystal photoluminescence and mechanism investigation

**Fig. 5:**
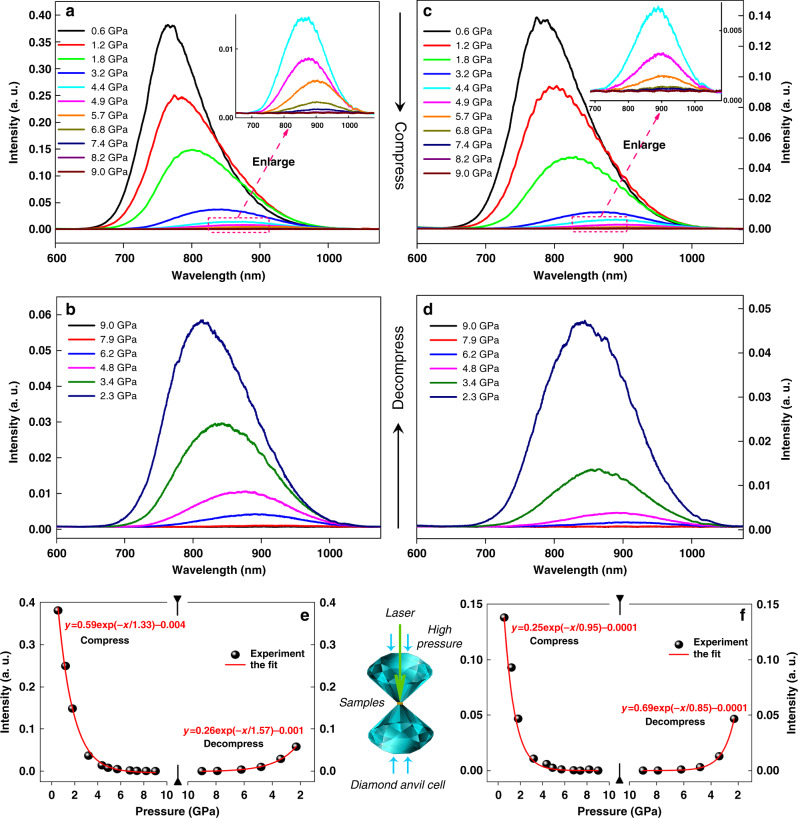
The pressure dependence of the PL spectra of Au_60_S_8*r*_ and Au_60_S_8*n*_. The PL spectra of Au_60_S_8*r*_ (**a**, **b**) and Au_60_S_8*n*_ (**c**, **d**) crystals upon the compression (**a**, **c**) and decompression (**b**, **d**); the nonlinear fit curves based on the experimental maximum intensity of Au_60_S_8*r*_ (**e**) and Au_60_S_8*n*_ (**f**) crystals upon the compression and decompression. The middle bottom shows the schematic diagram of a diamond anvil cell used in the high pressure experiments. Note: the measurement was performed on a laser scanning confocal Raman/PL microscope (HORIBA Jobin Yvon, λex = 532 nm, Power = 0.01 mW).

**Fig. 6:**
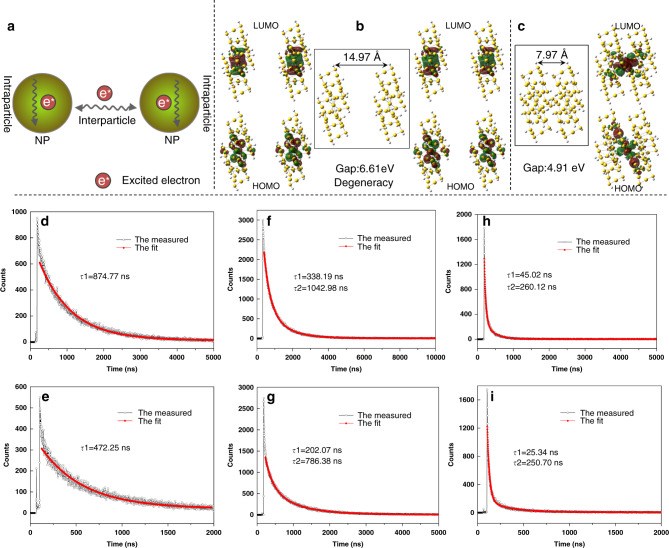
Illustration of the distance-dependent excitation electron transfer, and pressure-dependent HOMO-LUMO and PL decay profiles. (**a**) The interparticle and intraparticle non-radiative transfer of excited electrons; (**b**, **c**) the HOMO-LUMO distributions and gaps when the interparticle distances of Au_24_ are 14.97 (**b**) and 7.97 Å (**c**), respectively; the PL decay curves of Au_60_S_8*r*_ (**d**, **f**, **h**) and Au_60_S_8*n*_ (**e**, **g**, **i**) under different pressures: (**d**, **e**) atmospheric pressure; (**f**, **g**) 0.2 GPa; (**h**, **i**) 1.8 GPa. Note: 14.97 Å corresponds to the distance of two neighboring Au_24_ NCs in crystal.

Obtaining metal NC conformational isomer crystals provides an excellent opportunity for investigating crystallization-induced PL in depth. The PL intensity of Au_60_S_8*r*_ increases by ~60% with the maximum emission wavelength blueshifted by ~7 nm compared with that of Au_60_S_8n_ under similar conditions (Supplementary Fig. 16). The investigation on their absolute PL quantum yield (QY) also demonstrated that the Au_60_S_8*r*_ crystals (QY: 9.0%) is higher than that of Au_60_S_8*n*_ crystals (QY: 5.6%). Despite of different emission in crystals, they have the same emission in solution (see Supplementary Fig. 17), indicating that the conformational isomerism does not influence the solution emission herein. Although the more compact crystallographic packing can restrict the interparticle and intraparticle motion^8^, it also leads to stronger interaction of NCs, which might weaken the emission, and it is a dominant factor in determining the PL for some extreme cases. A simple and intuitive method to compare the interaction of NCs is the comparison of interparticle distance. A shorter interparticle distance means a stronger interaction^42,43^. For example, the average interparticle distance (2.20 nm) of Au_60_S_8*n*_ is shorter than that of Au_60_S_8*r*_ (2.34 nm, herein the interparticle distance was defined as the distance between two particle metal cores, see Supplementary Fig. 18), indicating a stronger interparticle interaction of Au_60_S_8*n*_ compared with that of Au_60_S_8*r*_. As far as we know, there is no experimental work concerning the structure and property tuning of metal NCs by way of high pressure, however, previous theoretical work^44^ has indicated that strain could affect the electronic band structure (band gap) of a ligand-protected gold cluster lattice, which provides a reference for this work. Herein to further verify our conclusion, high pressure was applied to the crystal samples (Supplementary Fig. 19) to reduce the interparticle distance and observe the resulting PL intensity change. Note that, the emission is excitation wavelength-dependent as shown in Fig. 5 and Supplementary Fig. 20. For comparison, the 532 nm wavelength was adopted throughout this manuscript. Indeed, it is demonstrated that, upon the compression, the emissions of both Au_60_S_8*r*_ and Au_60_S_8*n*_ obviously decrease, as shown in Fig. 5a, c. Of note, the maximum emission wavelength redshifts with the pressure increase, which also indicates that the interaction between the NCs was strengthened with the interparticle distance shortened. Up to ~9.0 GPa, the emission peaks almost disappear. Interestingly, the vanished emission can somehow restore upon the decompression (Fig. 5b, d and Supplementary Fig. 16), which indicates that the structures of the clusters are not essentially altered. The in situ crystal structure measurement confirms this. The pressure dependent XRD evolution of Au_60_S_8*n*_ crystal with every peak assigned was illustrated in Supplementary Fig. 21. The peak position “redshifts” upon compression correspond to the pressure-induced decrease of lattice constants and indicate the decrease of interparticle distance^45^, which was verified by the fact that the peak positions were restored under decompression, see Supplementary Fig. 21. The pressure dependences of the maximum intensity, the integrated intensity and the full width at half maximum (FWHM) are shown in Supplementary Fig. 22. The correlation between the pressure and the maximum PL intensity in a quantitative way was given in Fig. 5e, f, which demonstrate that the maximum PL intensity well conforms to the negative exponential function of the pressure during the investigated pressure range not only for the compression process but also for the decompression process. Note that, the emission can not recover at all after a higher pressure up to ~29.4 GPa was exerted (Supplementary Fig. 23), indicating that the crystal structure of the NCs essentially changes under such high pressure^46–48^. As a comparison, the PL spectra of both amorphous Au_60_S_8*r*_ and Au_60_S_8*n*_ are shown in Supplementary Fig. 24, which reveal the similar pressure-dependent trends as those of crystal samples. These facts unambiguously demonstrated that the crystalline PL depends on the interparticle distance. A question naturally arising is why the radiative decay was inhibited when the interparticle distance was reduced. The non-radiative decay by intraparticle and interparticle motion can be excluded since, in our case, more compactly arranged Au_60_S_8*n*_ with shorter interparticle distances should have less intraparticle and interparticle motion compared with the less compactly arranged Au_60_S_8*r*_, and the increase in pressure also restricts the motion of the NCs. However, with the decrease of interparticle distances, the interparticle interaction should increase, and even overlapping of the electron clouds of NCs can occur, which causes the decrease of HOMO-LUMO gap of NCs. As a result, the interparticle and intraparticle non-radiative transfers of excited electrons (Fig. 6a) accelerate^49–51^. The theoretical calculations provide strong support for this, see Fig. 6b, c and Supplementary Tables 1, 2. Note that, to save the computation cost, only two simplified Au_24_ were employed as the model. Experimental results also indicate the pressure-dependent PL of Au_24_, see Supplementary Fig. 25. The non-radiative excitation electron transfer can effectively deactivate the radiation energy and thus weaken the emission. This hypothesis can explain CIPW and can also interpret the well-known ACQ and AIE phenomena. When planar luminophores stack together by π···π interactions^8,52^, the short inter-luminophore distance enhances the non-radiative excitation electron transfer and thus leads to the quenching of PL. In the AIE case, the twisted structure (or steric hindrance) prevents the approach of luminophores, thus inhibiting the non-radiative excitation electron transfer between the neighboring luminophores. In addition, the aggregation of molecules also restricts the interparticle and intramolecular motion^8,52^. Consequently, the radiative decay content increases and results in extensive PL. Note that, in this hypothesis long inter-particles (luminophores) distance can NOT result in effective non-radiative excitation electron transfer between the neighboring particles (luminophores), and this transfer accelerates with the decrease of inter-particles (luminophores) distance in some range. The PL lifetime measurements provide another support for the hypothesis: under atmospheric pressure, there is only one lifetime for both conformers (874.77 vs. 472.25 ns, Au_60_S_8*r*_ vs. Au_60_S_8*n*_). Upon compression, the single lifetime turns to two ones for both cases, and the lifetimes shorten with the increases of pressures, see Fig. 6d-i, which might correspond to the fact that the inter-particle interaction contributes an additional lifetime and the lifetimes decrease with the decrease of energy gap originating from the increase of pressure, since it is known that the shorter lifetime correlates the narrower energy gap in some range with other conditions essentially untouched^44,53,54^. Note that, the concentration quenching or compress-induced quenching for organic luminophores was previously attributed to the forming of π–π stacking^11,55,56^, excimers/exciplexes^16,57,58^, trapping sites for excitation energy^59^, the inductive resonance energy transfer^15^, the polarization effect of adjacent molecules^60^, and some other reasons^61,62^. In most of the above mentioned cases, the PL quenching relates to the overlap of intermolecular planum structure. However, in our case, there are no such overlaps, and the intraparticle contribution for PL weakening was also considered.

## Discussion

In summary, we synthesized a NC by single sulfur doping of Au_60_S_7_(SCH_2_Ph)_36_ and characterized the as-obtained Au_60_S_8_(SCH_2_Ph)_36_ by ESI-MS and SCXC. Interestingly, we isolated two types of crystals (rectangular vs*.* needle-like), which have different NC conformations and arrangements, as determined by SCXC. The isolation of conformational isomers from a product mixture has not been previously reported. In addition, we introduce the concept of conformational isomerism into the field of nanochemistry. Furthermore, we revealed that the two conformational isomers can be converted to each other by regulating the crystallization solvent. Obtaining conformational isomers provides an excellent opportunity for investigating crystalline PL in depth. Indeed, the PL comparison between the two isomers indicates that the shortening of the interparticle distance weakens the emission of the NCs, which was further supported by the fact that the maximum PL intensity conformed to the negative exponential function of the pressure during the investigated pressure range. On the basis of these facts, we proposed an excitation electron transfer model to interpret crystallization-induced PL weakening, which was further supported by theoretical calculations and lifetime measurements. The hypothesis can explain both ACQ and AIE phenomena, too. Another point of this work is that high pressure is shown to be a powerful tool in the NC field, which may trigger more studies on the high-pressure physics and chemistry of metal NCs in the future (for example, exploiting high pressure for structure and property tuning of metal NCs).

## Methods

### Synthesis of Au_60_S_8_ NCs

Typically, 10 mg of Au_60_S_7_ NCs was dissolved in 0.5 ml of HSCH_2_Ph. The reaction proceeded overnight with stirring at 100 °C, and then it was terminated by the addition of methanol. The crude product was thoroughly washed with methanol four times and then subjected to subsequent separation and purification by PTLC. Note that, Au_60_S_7_ NCs were prepared according to our previous report^39^.

### Single crystal growth

Single crystals of the purified NCs were grown by vapor diffusion of acetonitrile into a benzene solution for one month. Typically, ~ 3 mg of the purified NCs were dissolved in 3 ml of benzene. Then, the NC solution was placed in a 20 ml of bottle containing 12 ml of acetonitrile. After one month, the rectangular and needle-like crystals were obtained by vapor diffusion of acetonitrile into the benzene solution. Moreover, by adjusting the amount of acetonitrile and benzene solution, the amount of the rectangular and needle-like crystals can be tuned.

### Theoretical calculation

The quantum chemical computations were carried out by the Gaussian 16 program (Revision B01)^63^. The theoretical method is the functional B97X-D, including the empirical dispersion^64^. The turbomole series Def2-SVP basis set was used for the H, S, and Au atoms. Specially, for the heavy metal Au atom, the effective core potential (ECP) included in the Def2-SVP basis set was used to reduce the computational cost^65^.

For the Au_24_(SCH_2_Ph)_20_ (Au_24_) NCs, we use the crystal structure from the experiment, and replace the CH_2_Ph group by the hydrogen atom for simplifying the computation. After the replacing, all hydrogen atoms have been optimized with the Au_24_S_20_ core fixed. Note that, Au_24_ NCs were prepared according to our previous report^66^.

### Characterization

The single crystal diffraction data of Au_60_S_8*r*_ and Au_60_S_8*n*_ were recorded on a Bruker APEXDUO X-ray Diffractometer (Bruker, Germany). ESI-MS was conducted on a Waters Q-TOF mass spectrometer equipped with a Z-spray source, and the source temperature was kept at 70 °C. To prepare the samples for ESI-MS analysis, Au_60_S_8*r*_ or Au_60_S_8*n*_ was dissolved in toluene (~0.5 mg/ml) and then diluted (1/1, V/V) with an ethanol solution containing 0.5 mM CsOAc. The sample was directly infused into the chamber at 5 μl/min. The spray voltage is 2.20 kV, and the cone voltage is kept at 60 V.  The solution PL spectra of Au_60_S_8*r*_ and Au_60_S_8*n*_ were recorded on a Fluorolog-3-21 (HORIBA Jobin Yvon) with a xenon lamp as the excitation source and the excitation wavelength was kept at 514 nm (OD_514_ ~ 0.1) with a slit of 10 nm. The diamond anvil cell was prepared by preindenting the stainless steel gasket to a thickness of ~100 μm from 250 μm through which an ~200 μm diameter hole was drilled and served as the sample chamber. The Au_60_S_8*r*_ and Au_60_S_8*n*_ were loaded with two ruby chips to calibrate the pressure by laser-excited ruby fluorescence during the in situ experiments. 4/1 (V/V) of methanol and ethanol was used as the transmitting pressure medium. The in situ high pressure PL spectra of Au_60_S_8*r*_, Au_60_S_8*n*_, and Au_24_(SCH_2_Ph)_20_ were recorded on a laser confocal scanning Raman/luminescence microscope (HORIBA Jobin Yvon) with a laser (532/633 nm) power of 0.01 mW. The spectrum was averaged by recording at three different positions under the same pressure. The absolute PL quantum yields of Au_60_S_8*r*_ and Au_60_S_8*n*_ crystals were conducted by UV/vis/NIR absolute PL quantum yield spectrometer (C13534, Quantaurus-QY Plus, HAMAMATSU). High-pressure XRD experiments with a wavelength of 0.6199 Å and a focused beam size of about 4 × 7 μm^2^ were performed at beamline 15U1, Shanghai Synchrotron Radiation Facility (SSRF), China. The PL lifetime of Au_60_S_8*r*_ and Au_60_S_8*n*_ at atmospheric and high pressure were recorded by an Edinburgh FLS980 lifetime and steady state spectrometer using a 470 nm pulse laser. The PTLC plates were eluted with dichloromethane/petroleum ether mixture (1/1, V/V) at room temperature under air atmosphere.

## Supplementary information

Supplementary Information

## Data Availability

The X-ray crystallographic coordinates for structures reported in this study (see Supplementary Table 3, 4) have been deposited at the Cambridge Crystallographic Data Centre (CCDC), under deposition numbers CCDC 2026360 for Au_60_S_8*r*_ and CCDC 2026358 for Au_60_S_8*n*_. These data can be obtained free of charge from The Cambridge Crystallographic Data Centre via www.ccdc.cam.ac.uk/data_request/cif. Other data are also available from the authors on reasonable request.
