# Supplementary material for: Distance makes a difference in crystalline photoluminescence
Source: Nat Commun. 2020 Nov 4;11:5572. doi: 10.1038/s41467-020-19377-6 (PMC7643180; doi:10.1038/s41467-020-19377-6)
Supplement: Supplementary file 1 — Supplementary Information [file 41467_2020_19377_MOESM1_ESM.pdf]

Supplementary Information for

Distance makes a difference in crystalline photoluminescence

Zibao Gan, Yungui Liu, Lin Wang, Shuqing Jiang, Nan Xia, Zhipeng Yan, Xiang Wu, Junran Zhang, Wanmiao Gu, Lizhong He, Jingwu Dong, Xuedan Ma, Jaeyong Kim, Zhongyan Wu, Yixuan Xu, Yanchun Li and Zhikun Wu*

*Correspondence to: zkwu@issp.ac.cn.

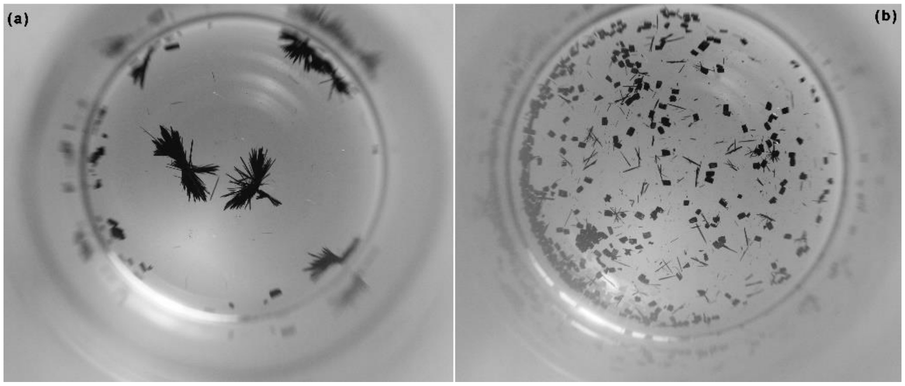

Supplementary Figure 1. Photographs of the as-obtained single crystals in various culture solvents. (a) 3 mL of benzene and 6 mL of acetonitrile, (b) 3 mL of benzene and 12 mL of acetonitrile (Note: the needle-like and rectangular single crystals are concurrently obtained in both a and b systems, but their contents are case-dependent).

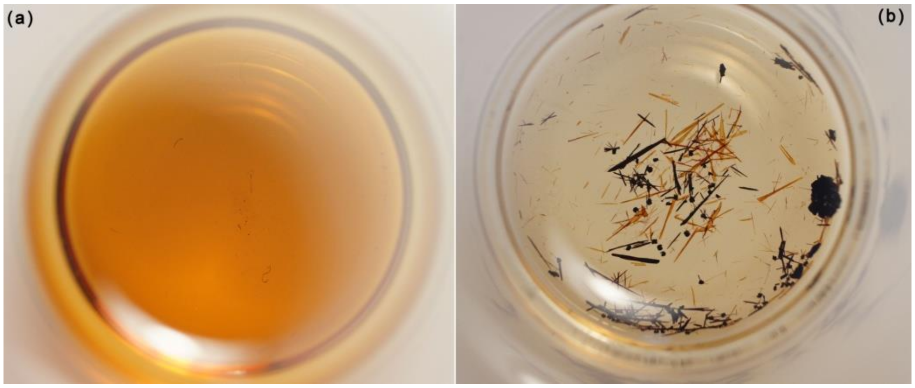

Supplementary Figure 2. Photographs of the as-obtained single crystals in various culture solvents. (a) 3 mL of benzene without acetonitrile, (b) 3 mL of benzene and 3 mL of acetonitrile (Note that, the crystal growth time for a is ~ 3 months and for b is ~ one month, respectively).

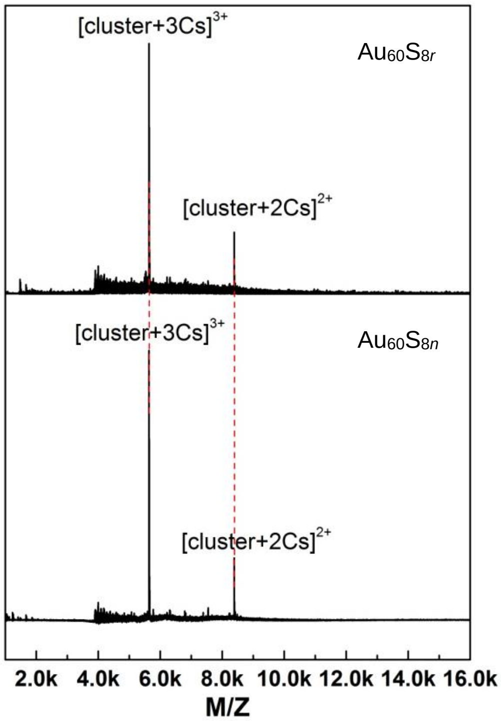

Supplementary Figure 3. ESI-MS (acquired in positive ion mode) of $Au_{60}S_{8r}$ and $Au_{60}S_{8n}$.

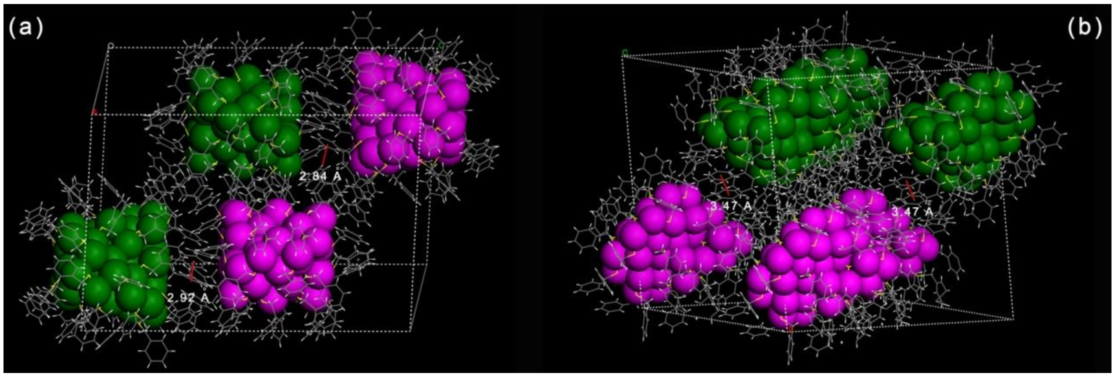

Supplementary Figure 4. The unit cell of (a) $Au_{60}S_{8r}$ and (b) $Au_{60}S_{8n}$. Color labels: the gold atoms in enantiomers are respectively shown in green and magenta.

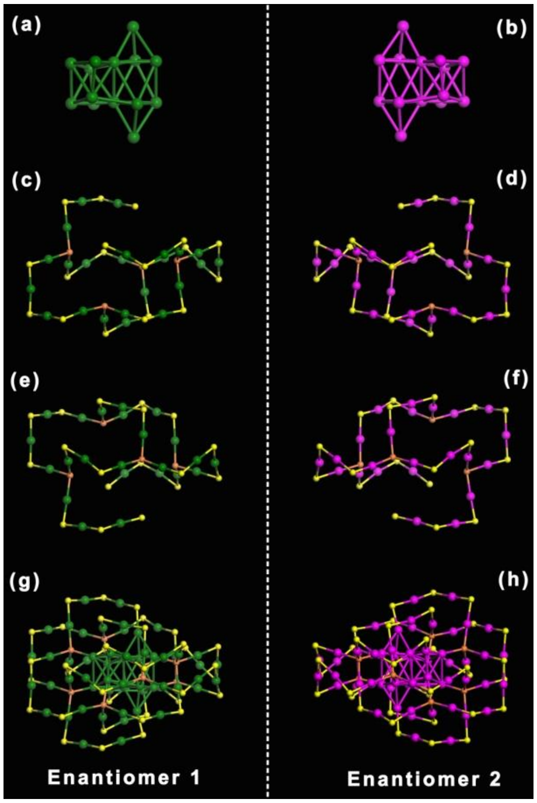

Supplementary Figure 5. The anatomy of the two enantiomers in the unit cell of $Au_{60}S_{8r}$ crystal: a, b) Au_{14} kernel; c-f) $Au_{23}S_4(SCH_2Ph)_{18}$ staples; g, h) the overall framework. For clarity, C and H are omitted. S yellow, Au green or magenta.

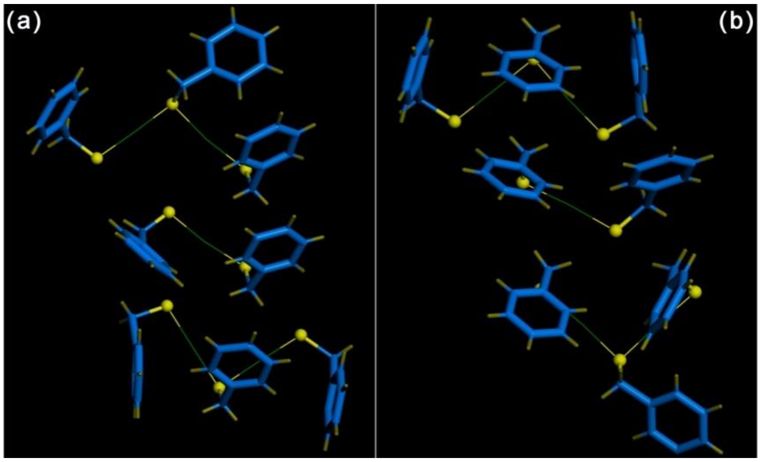

Supplementary Figure 6. Down view of 8 phenylmethanethiolate ligands constituting the foot in (a) $Au_{60}S_{8r}$ *vs* (b) $Au_{60}S_{8n}$ nanocluster. Color labels: Au atoms in green, S atoms in yellow.

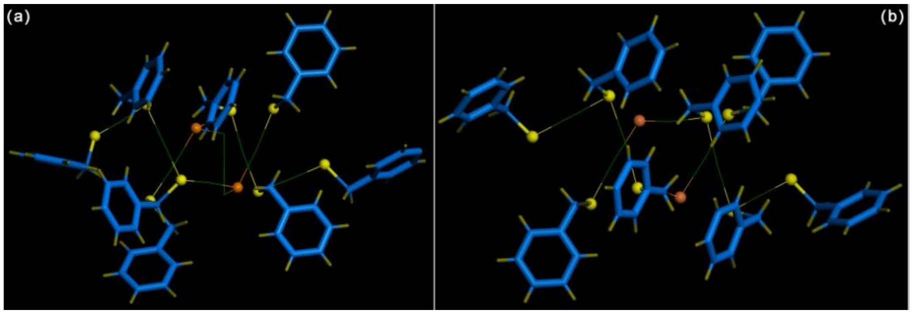

Supplementary Figure 7. Side view of 8 phenylmethanethiolate ligands constituting the torso in (a) $Au_{60}S_{8r}$ *vs* (b) $Au_{60}S_{8n}$ nanocluster. Color labels: Au atoms in green, the μ_4-S atoms in orange, other S atoms in yellow.

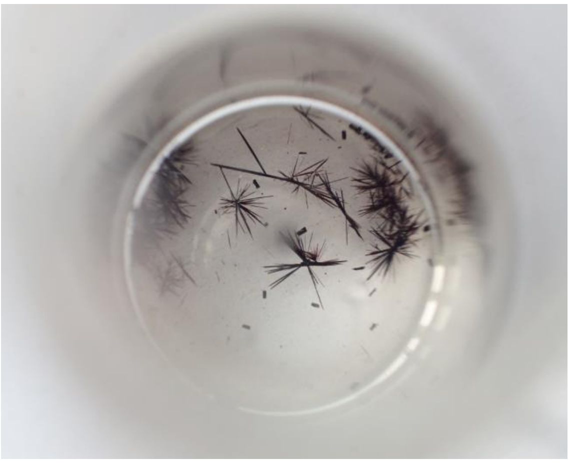

Supplementary Figure 8. Photograph of the recrystallized result of the dissolved rectangular crystals in a system of 3 mL of benzene and 6 mL of acetonitrile.

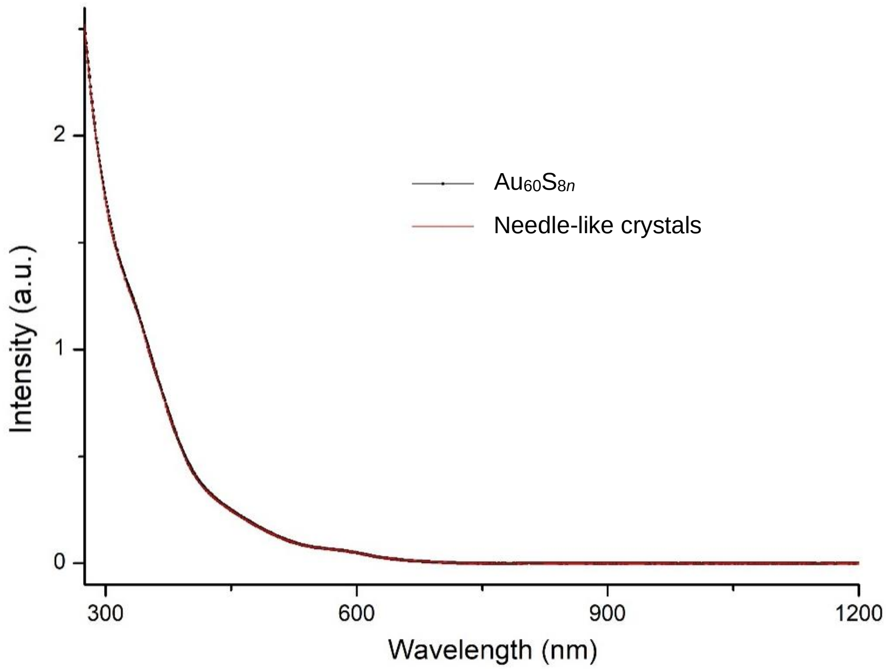

Supplementary Figure 9. UV/vis/NIR absorption spectra of $Au_{60}S_{8n}$ and needle-like crystals obtained from $Au_{60}S_{8r}$.

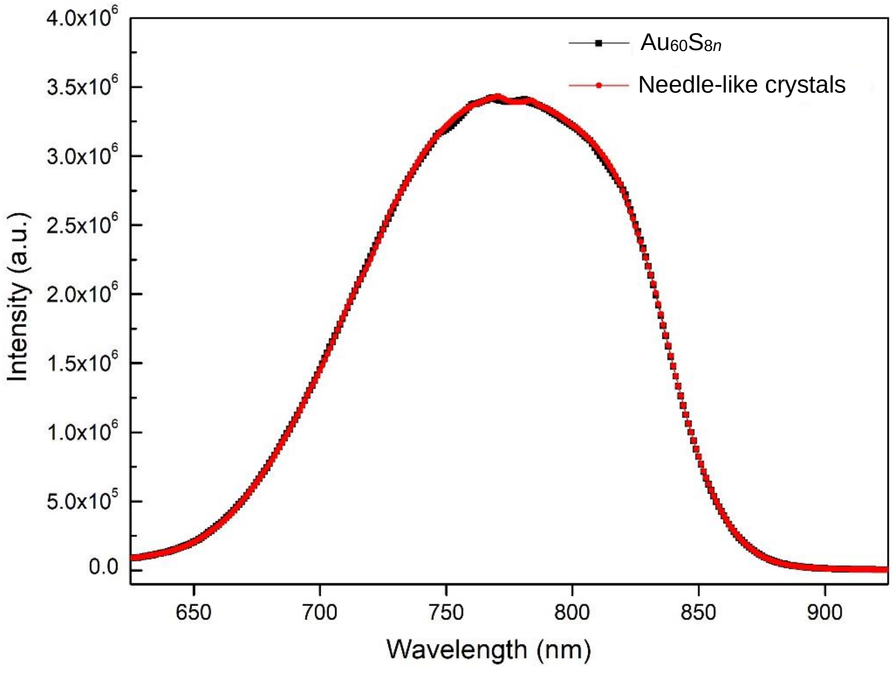

Supplementary Figure 10. Solution PL of $Au_{60}S_{8n}$ and needle-like crystals obtained from $Au_{60}S_{8r}$. Note: the excitation wavelength -514nm, OD_{514} ~ 0.1, dichloromethane as solvent.

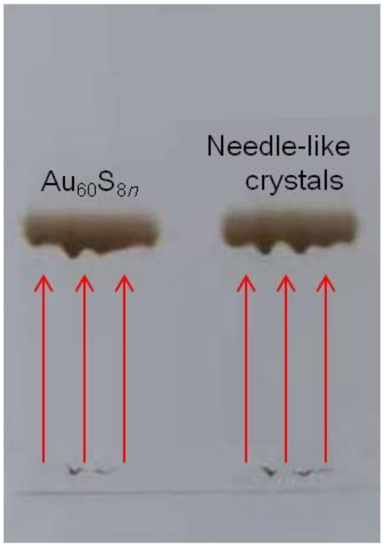

Supplementary Figure 11. PTLC results of $Au_{60}S_{8n}$ and needle-like crystals obtained from $Au_{60}S_{8r}$.

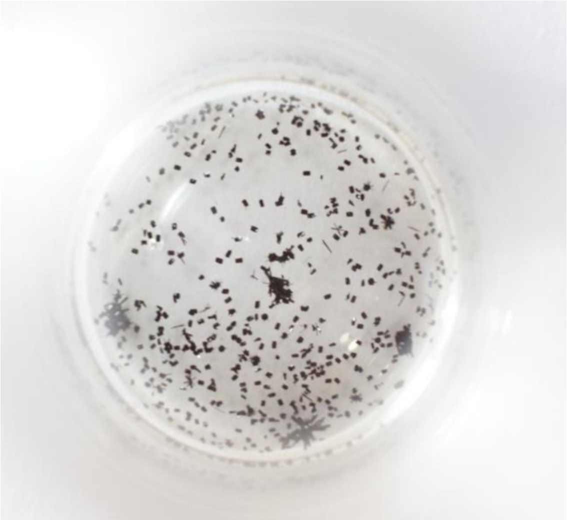

Supplementary Figure 12. Photograph of the recrystallized result of the dissolved needle-like crystals in a system of 3 mL of benzene and 12 mL of acetonitrile.

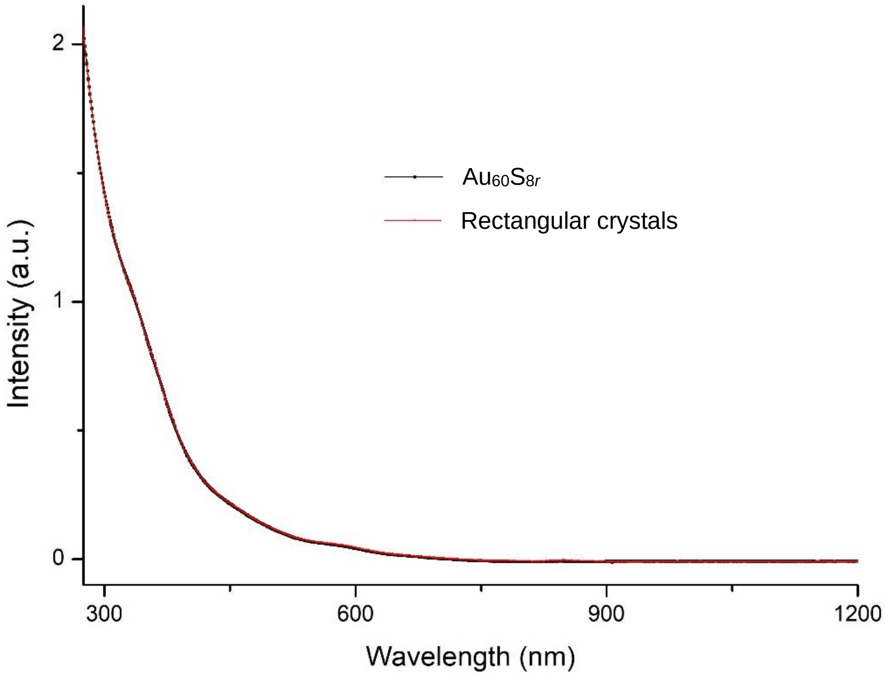

Supplementary Figure 13. UV/vis/NIR absorption spectra of $Au_{60}S_{8r}$ and rectangular crystals obtained from $Au_{60}S_{8n}$.

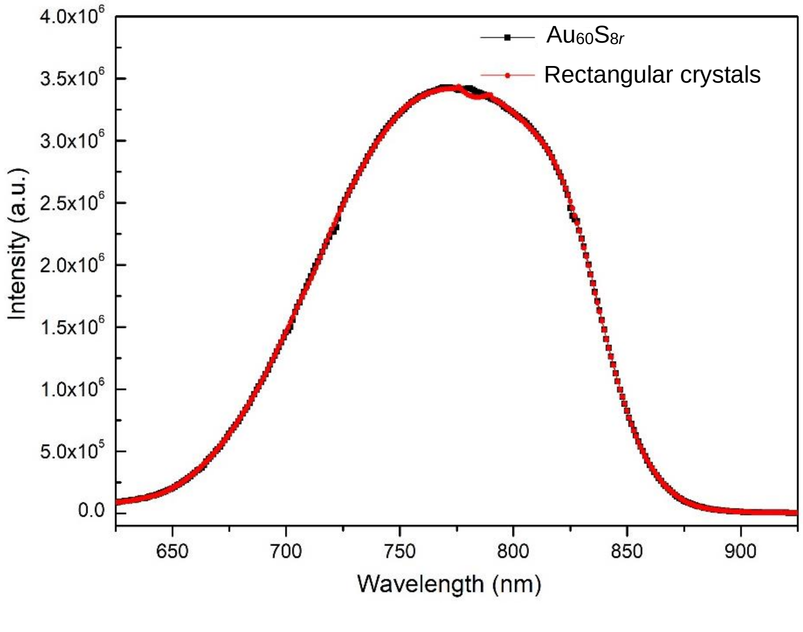

Supplementary Figure 14. Solution PL of $Au_{60}S_{8r}$ and rectangular crystals obtained from $Au_{60}S_{8n}$. Note: the excitation wavelength -514 nm, OD_{514} ~ 0.1, dichloromethane as solvent.

.

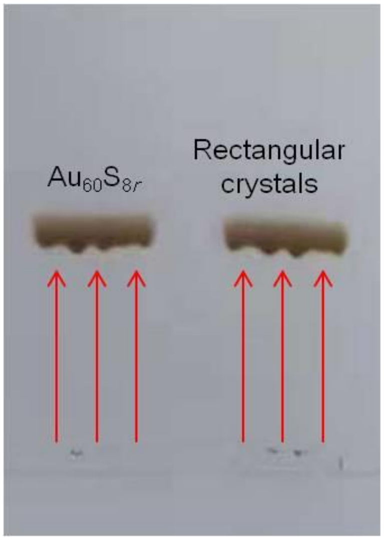

Supplementary Figure 15. PTLC results of $Au_{60}S_{8r}$ and rectangular crystals obtained from $Au_{60}S_{8n}$.

.

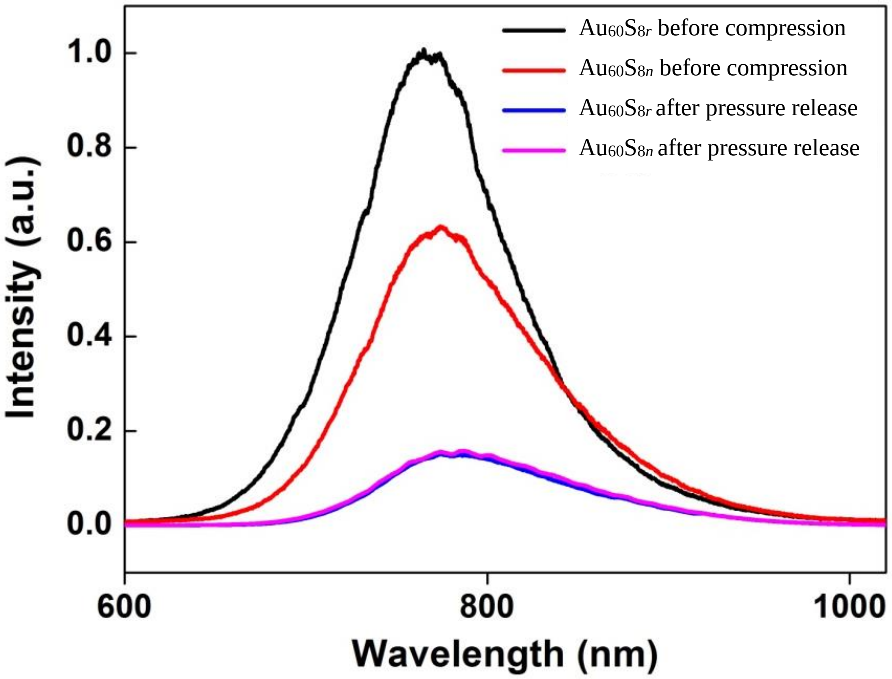

Supplementary Figure 16. PL spectra $Au_{60}S_{8r}$ and $Au_{60}S_{8n}$ crystals before compression and after pressure release.

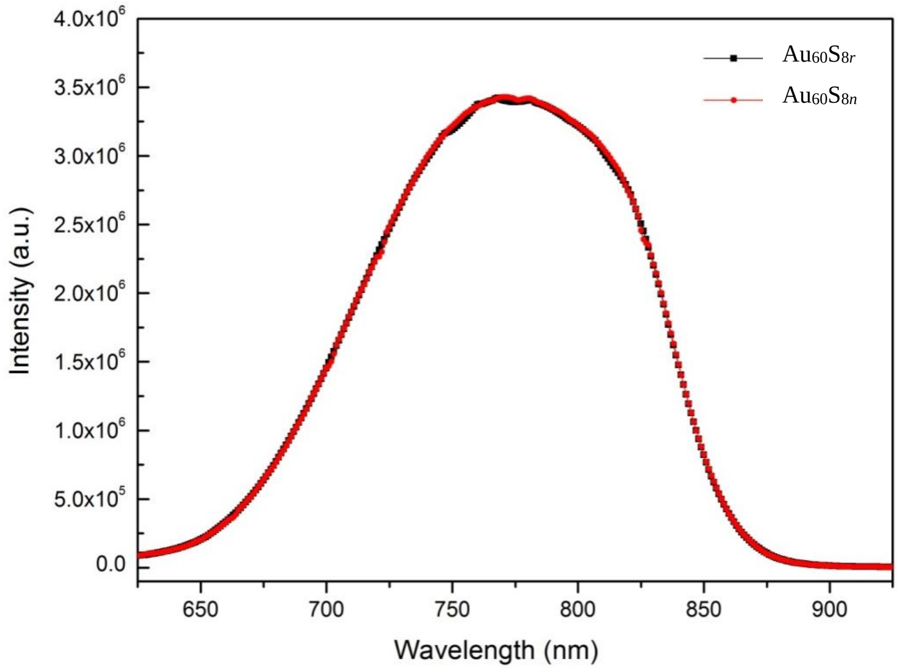

Supplementary Figure 17. Solution PL of $Au_{60}S_{8r}$ and $Au_{60}S_{8n}$. Note: the excitation wavelength - 514nm, OD_{514} ~ 0.1, dichloromethane as solvent.

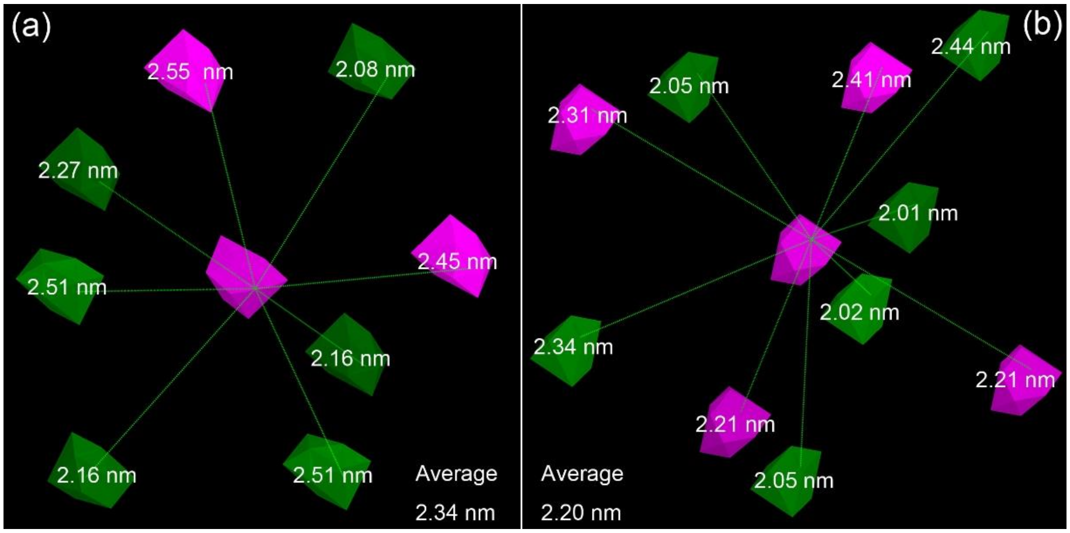

Supplementary Figure 18. Interparticle distance of (a) $Au_{60}S_{8r}$ and (b) $Au_{60}S_{8n}$ in crystal. Color labels: the kernel gold atoms in enantiomers are respectively shown in magenta and green.

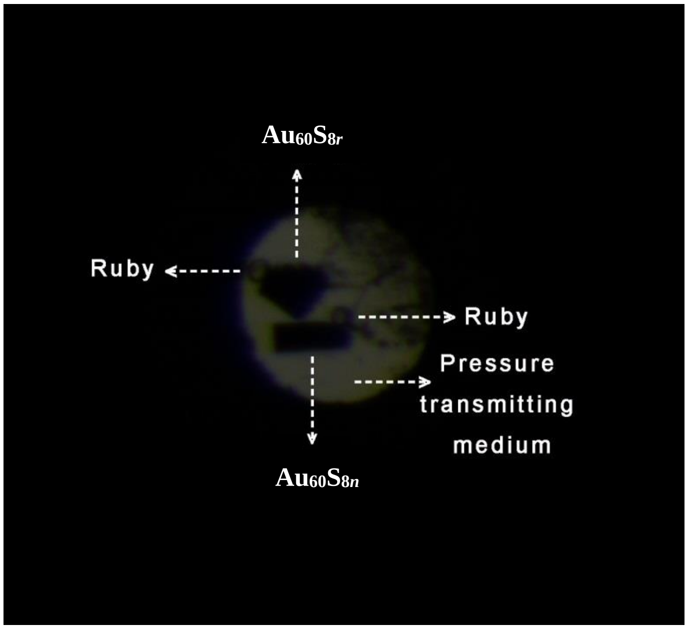

Supplementary Figure 19. Photograph of the $Au_{60}S_{8r}$ and (b) $Au_{60}S_{8n}$ crystals in diamond anvil cell.

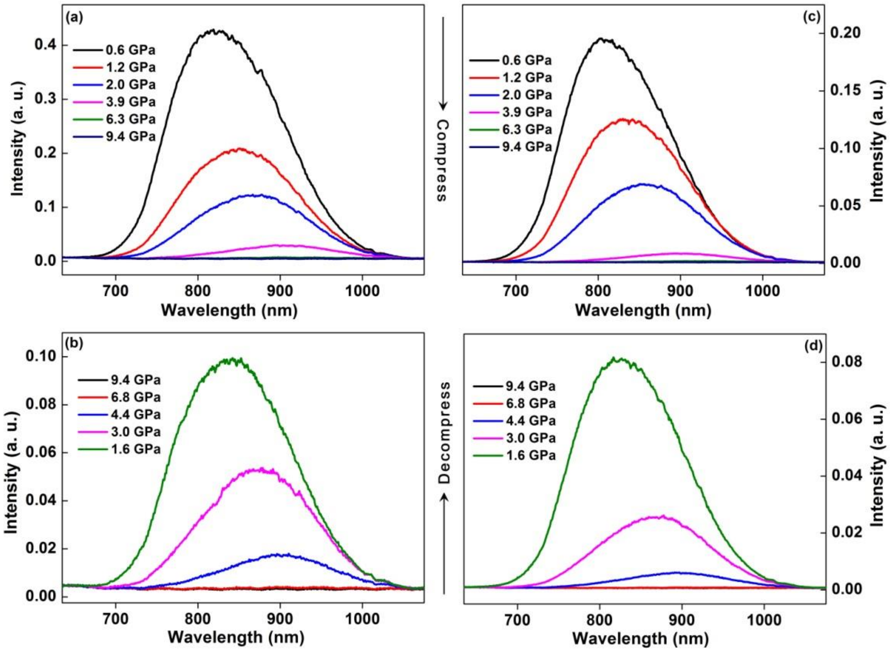

Supplementary Figure 20. The pressure dependence of the PL spectra of $Au_{60}S_{8r}$ (a, b) and $Au_{60}S_{8n}$ (c, d) crystals upon the compression and decompression. Note: the measurement was performed on a laser scanning confocal Raman/PL microscope (HORIBA Jobin Yvon, λex = 633 nm, Power = 0.01 mW).

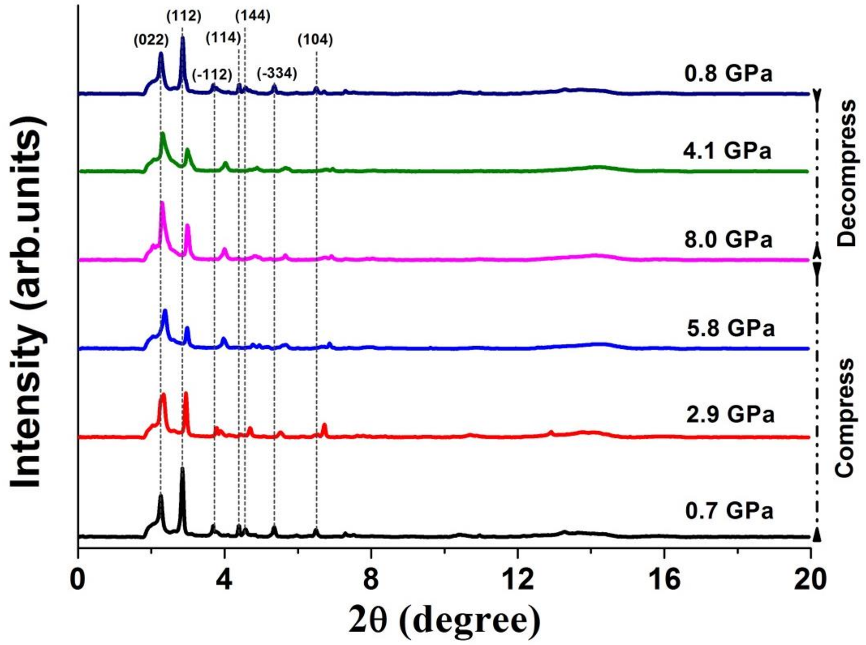

Supplementary Figure 21. The pressure dependence of XRD of $Au_{60}S_{8n}$ crystal upon the compression and decompression (Note: only single angle scan can be conducted during high pressure XRD measurements, λ=0.6199 Å).

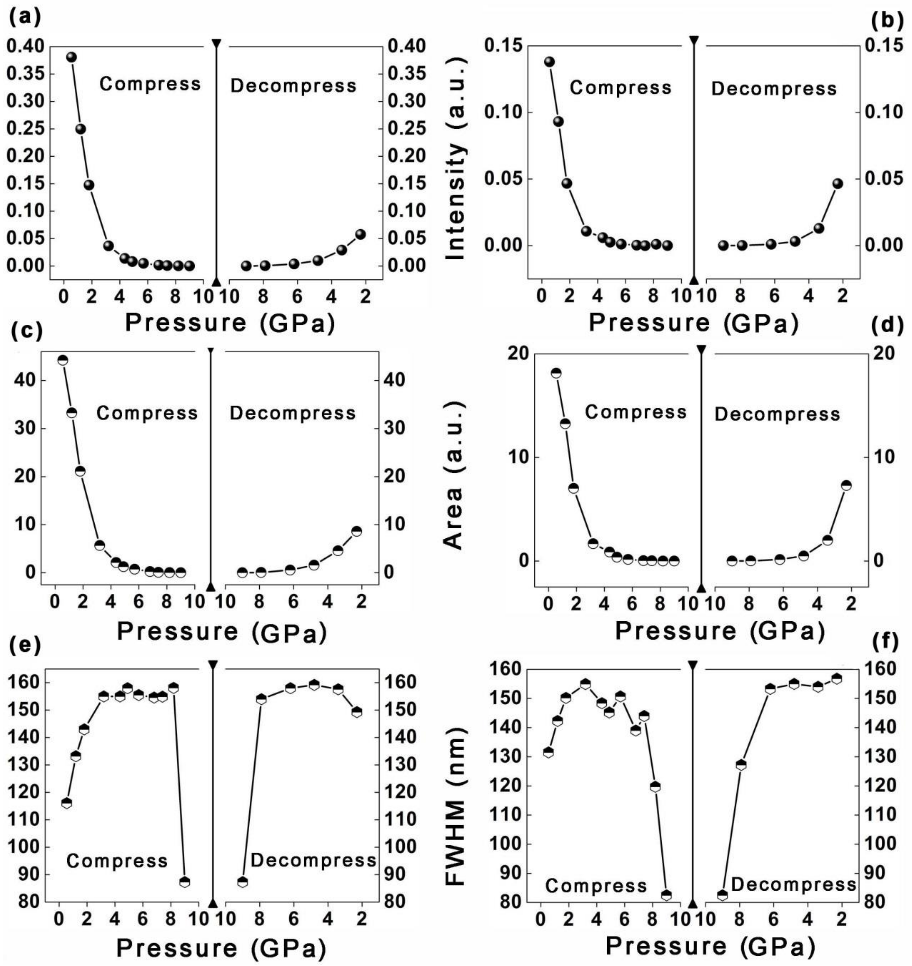

Supplementary Figure 22. The pressure dependence of the maximum intensity, the integrated intensity and the full width at half maximum (FWHM) of $Au_{60}S_{8r}$ and $Au_{60}S_{8n}$. The maximum intensity (a), the integrated intensity (c) and the full width at half maximum (e) of $Au_{60}S_{8r}$ crystal upon the compression and decompression; the maximum intensity (b), the integrated intensity (d) and the full width at half maximum (f) of $Au_{60}S_{8n}$ crystal upon the compression and decompression.

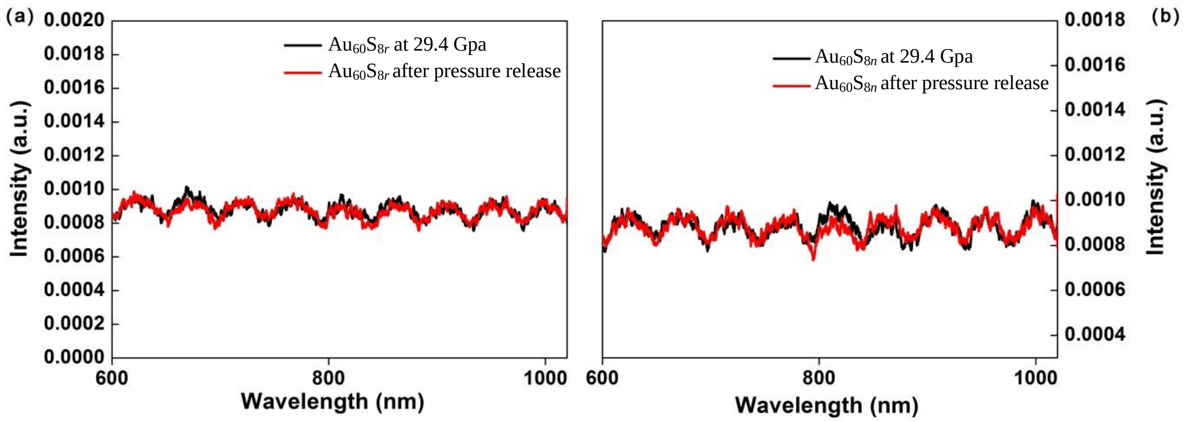

Supplementary Figure 23. (a-b) PL spectra of $Au_{60}S_{8r}$ and $Au_{60}S_{8n}$ crystals upon compression (29.4 GPa) and decompression.

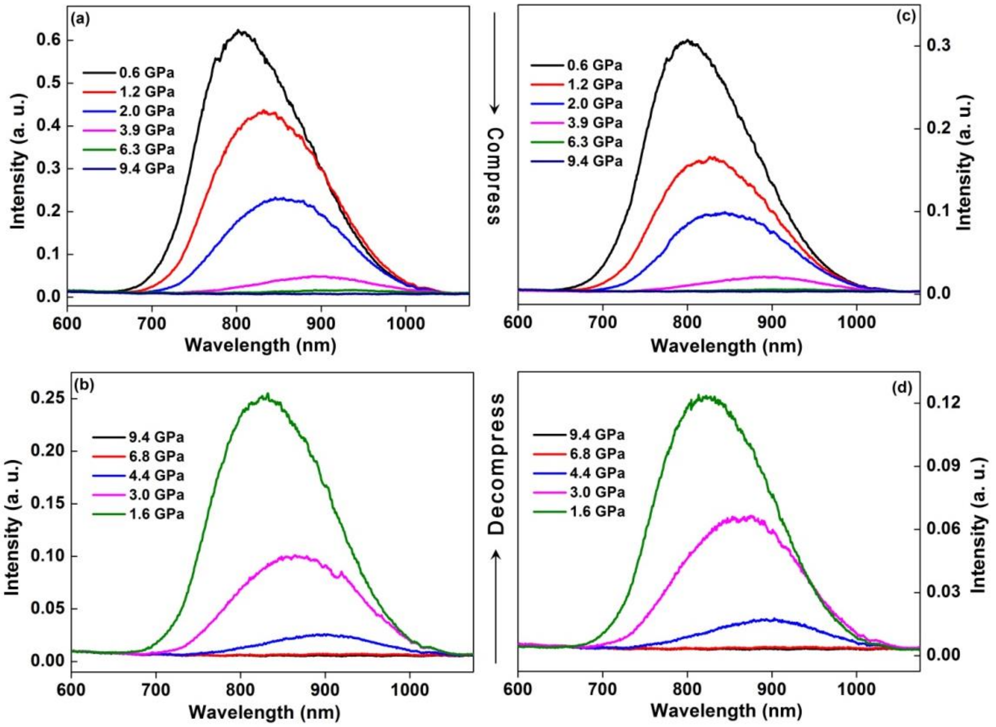

Supplementary Figure 24. The pressure dependence of the PL spectra of amorphous (a, b) $Au_{60}S_{8r}$ and (c, d) $Au_{60}S_{8n}$ samples upon the compression and decompression. Note: the measurement was performed on a laser scanning confocal Raman/PL microscope (HORIBA Jobin Yvon, λex = 532 nm, Power = 0.01 mW).

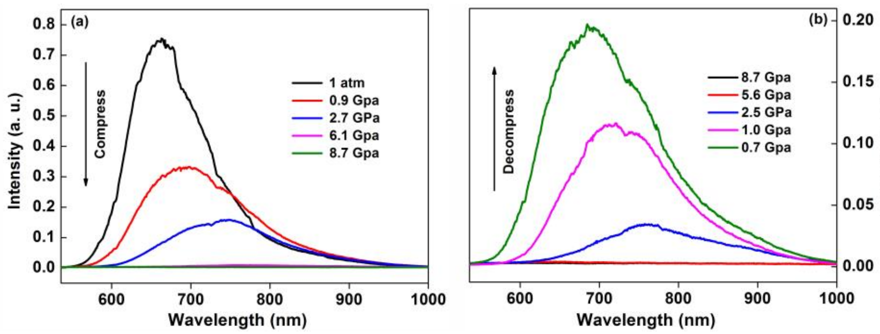

Supplementary Figure 25. The pressure dependence of PL spectra of Au_{24} crystal upon the compression (a) and decompression (b). Note: the measurement was performed on a laser scanning confocal Raman/PL microscope (HORIBA Jobin Yvon, λex = 532 nm, Power = 0.01 mW).

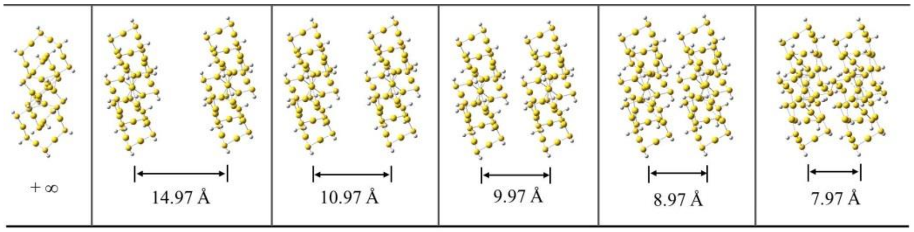

Interparticle distance (Å)	HOMO (eV)	LUMO (eV)	HOMO-LUMO gap (eV)
+ ∞	-7.84	-1.23	6.61
14.97	-7.83	-1.22	6.61
10.97	-7.81	-1.21	6.60
9.97	-7.78	-1.22	6.56
8.97	-7.03	-1.27	5.76
7.97	-6.35	-1.44	4.91

Supplementary Table 1. The interparticle distance-dependent HOMO-LUMO energies of Au_{24}. Note: When the interparticle distance is very far (+ ∞), the interparticle interaction can be neglected; 14.97 Å corresponds to the distance of two neighboring Au_{24} nanoclusters in crystals.

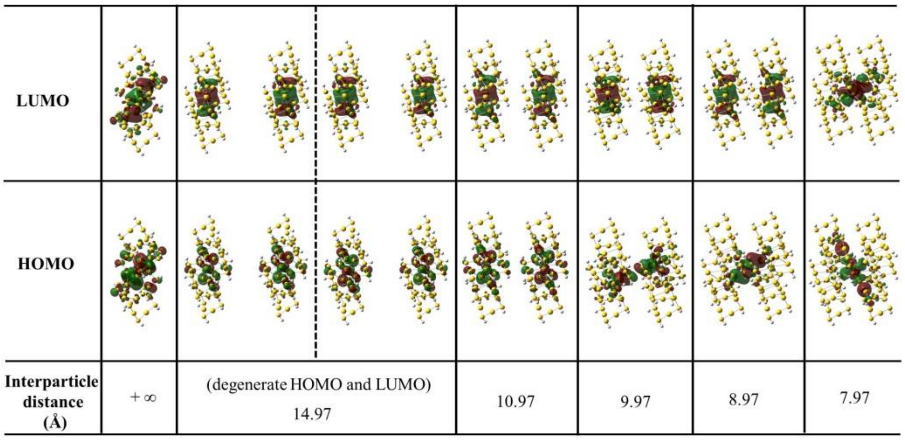

Supplementary Table 2. The interparticle distance-dependent HOMO-LUMO distributions of Au_{24}. Note: When the interparticle distance is very far (+ ∞), the interparticle interaction can be neglected; 14.97 Å corresponds to the distance of two neighboring Au_{24} nanoclusters in crystals.

Supplementary Table 3. Crystal data and structure refinement for $Au_{60}S_{8r}$.

Empirical formula	$C_{252}H_{252}Au_{60}S_{44}$
Formula weight	16509.15
Temperature	296(2) K
Wavelength	0.71073 Å
Crystal system	Monoclinic
Space group	P21/c
Unit cell dimensions	a = 37.010(2) Å α = 90°.
	b = 26.4334(14) Å β = 104.880(2)°.
	c = 40.268(3) Å γ = 90°.
Volume	38073(4) $Å^3$
Z	4
Density (calculated)	2.880 g/cm^3
Absorption coefficient	23.286 mm^{-1}
F(000)	28832
Crystal size	0.200 x 0.200 x 0.200 mm^3
Theta range for data collection	2.023 to 25.102°.
Index ranges	-44<=h<=35, -30<=k<=31, -48<=l<=37
Reflections collected	176201
Independent reflections	67277 [R(int) = 0.0573]
Completeness to theta = 25.102°	99.2 %
Refinement method	Full-matrix least-squares on F^2
Absorption correction	Semi-empirical from equivalents
Data / restraints / parameters	67277 / 69403/ 3477
Goodness-of-fit on F^2	1.039
Final R indices [I>2sigma(I)]	R1 = 0.0863, wR2 = 0.2385
R indices (all data)	R1 = 0.1394, wR2 = 0.2909
Extinction coefficient	n/a
Largest diff. peak and hole	6.552 and -2.859 e.$Å^{-3}$

Supplementary Table 4. Crystal data and structure refinement for $Au_{60}S_{8n}$.

Empirical formula	$C_{252}H_{252}Au_{60}S_{44}$	
Formula weight	16509.15	
Temperature	296(2) K	
Wavelength	0.71073 Å	
Crystal system	Monoclinic	
Space group	C2/c	
Unit cell dimensions	a = 28.092(2) Å	α= 90°.
	b = 39.268(4) Å	β= 100.534(2)°.
	c = 32.507(3) Å	γ= 90°.
Volume	35255(5) $Å^3$	
Z	4	
Density (calculated)	3.110 g/cm^3	
Absorption coefficient	25.148 mm^{-1}	
F(000)	28832	
Crystal size	0.230 x 0.050 x 0.050 mm^3	
Theta range for data collection	2.22 to 30.14°.	
Index ranges	-28<=h<=39, -55<=k<=54, -45<=l<=45	
Reflections collected	179977	
Independent reflections	51848 [R(int) = 0.0579]	
Completeness to theta = 25.000°	99.6 %	
Absorption correction	Multi-Scan	
Max. and min. transmission	0.0232 and 0.0023	
Refinement method	Full-matrix least-squares on F^2	
Data / restraints / parameters	51848 / 22009 / 1862	
Goodness-of-fit on F^2	1.042	
Final R indices [I>2sigma(I)]	R1 = 0.0782, wR2 = 0.2072	
R indices (all data)	R1 = 0.1170, wR2 = 0.2397	
Extinction coefficient	n/a	
Largest diff. peak and hole	14.504 and -4.419 e.$Å^{-3}$	
